# New Insights into Oxidative Stress and Inflammatory Response in Neurodegenerative Diseases

**DOI:** 10.3390/ijms25052698

**Published:** 2024-02-26

**Authors:** Eveljn Scarian, Camilla Viola, Francesca Dragoni, Rosalinda Di Gerlando, Bartolo Rizzo, Luca Diamanti, Stella Gagliardi, Matteo Bordoni, Orietta Pansarasa

**Affiliations:** 1 Cellular Models and Neuroepigenetics Unit, IRCCS Mondino Foundation, Via Mondino 2, 27100 Pavia, Italy; eveljn.scarian@mondino.it (E.S.); camilla.viola@mondino.it (C.V.); matteo.bordoni@mondino.it (M.B.); orietta.pansarasa@mondino.it (O.P.); 2 Department of Brain and Behavioral Sciences, University of Pavia, Via Agostino Bassi 21, 27100 Pavia, Italy; 3 Department of Biology and Biotechnology “L. Spallanzani”, University of Pavia, Via Adolfo Ferrata, 9, 27100 Pavia, Italy; francesca.dragoni@mondino.it (F.D.); rosalinda.digerlando@mondino.it (R.D.G.); 4 Molecular Biology and Transcriptomics Unit, IRCCS Mondino Foundation, Via Mondino 2, 27100 Pavia, Italy; bartolo.rizzo@mondino.it; 5 Neuroncology Unit, IRCCS Mondino Foundation, Via Mondino 2, 27100 Pavia, Italy; luca.diamanti@mondino.it

**Keywords:** oxidative stress, neurodegenerative diseases, amyotrophic lateral sclerosis, reactive oxygen species, inflammation

## Abstract

Oxidative stress (OS) and inflammation are two important and well-studied pathological hallmarks of neurodegenerative diseases (NDDs). Due to elevated oxygen consumption, the high presence of easily oxidizable polyunsaturated fatty acids and the weak antioxidant defenses, the brain is particularly vulnerable to oxidative injury. Uncertainty exists over whether these deficits contribute to the development of NDDs or are solely a consequence of neuronal degeneration. Furthermore, these two pathological hallmarks are linked, and it is known that OS can affect the inflammatory response. In this review, we will overview the last findings about these two pathways in the principal NDDs. Moreover, we will focus more in depth on amyotrophic lateral sclerosis (ALS) to understand how anti-inflammatory and antioxidants drugs have been used for the treatment of this still incurable motor neuron (MN) disease. Finally, we will analyze the principal past and actual clinical trials and the future perspectives in the study of these two pathological mechanisms.

## 1. Introduction

Neurodegenerative diseases (NDDs) are a group of age-related disorders, which cause the death of neural cells of specific types [1]. They are more common in the community as the average age of the population rises, becoming a serious worldwide diffuse health problem [2]. Annual incidence rates of NDDs are typically approximated at 10 to 15 per 100,000 people worldwide [3]. The diagnosis of NDDs is challenging because of the variety of clinical signs and symptoms and it is frequently only confirmed with a neuropathological investigation following the patient’s passing [4]. Despite their various symptoms, due to the distinct cell types and areas of the nervous system affected, they all manifest in two forms, a familial form and a sporadic one, not correlated with a familial history of the disease [1]. They share many pathological processes, including dysfunctions in the autophagosomal, ubiquitin/proteasomal, and lysosomal systems, proteins misfolding, and their aggregation. Moreover, they are all characterized by oxidative stress (OS) and neuroinflammation [5,6,7]. In this review, we will focus on the most common NDDs, Alzheimer’s disease (AD), Parkinson’s disease (PD), Huntington’s disease (HD), and amyotrophic lateral sclerosis (ALS), as well as on the role of OS and neuroinflammation in these diseases. Furthermore, we will dig deeper into ALS and the use of antioxidant and anti-inflammatory drugs in its therapy.

## 2. Oxidative Stress

The term OS was first formulated in 1985 [8] and refers to an imbalance between oxidants and antioxidants in a biological system, either because of the presence of elevated levels of reactive oxygen species (ROS) or due to a deficient function of the antioxidant system [6]. In a metabolic system there is a redox balance: physiological deviations from this balance are referred to as “oxidative eustress”, whereas non-physiological deviations are defined as “oxidative distress” [9]. Although oxygen is essential for human life, because of its structure and the presence of two unpaired electrons, it has the tendency to form radicals, including ROS. ROS are a class of oxygen-derived reactive molecules with a short-term life and a high reactivity [6] and include superoxide anions (O^2−^), hydroxyl radical (OH), hydrogen peroxide (H_2_O_2_), nitric oxide (NO), and lipid radicals [10]. ROS can have both an exogenous and an endogenous production. Exogenous sources include specific pharmaceuticals, ionizing radiations, and the metabolism of environmental chemicals, whereas endogenous sources are mitochondrial or non-mitochondrial ROS-developing enzymes [11]. In healthy cells, more than 90% of ROS are produced when an electron escapes from the electron transport chain in mitochondria and attaches to oxygen, whereas 10% of ROS are produced by enzymes, including dihydroorotate dehydrogenase, monoamine oxidase, and nicotinamide adenine dinucleotide phosphate oxidase [12,13]. Due to elevated oxygen consumption, in the brain, there is a high production of ROS, mainly due to electron transport chain complex 1, which contributes to the neurodegeneration of cells modulating important biomolecules, including DNA, RNA, proteins, lipids, and pathways, such as nucleic acid oxidation and lipid peroxidation [6]. Moreover, it was widely demonstrated that ROS can damage mitochondria affecting their proteins, lipids, and DNA, hampering their functions and leading to various diseases [14,15,16]. In complex organisms, such as humans, lipid and protein oxidation are more relevant than DNA oxidation, especially regarding NDDs, in which oxidized proteins acquire a toxic function and aggregate [13]. Also noteworthy is the nitrosative stress, which refers to combined biochemical reactions of NO and O^2−^, with the production of peroxynitrite anions. Peroxynitrite anions, in turn, can lead to the nitration of proteins, lipids, and DNA, affecting the enzyme activity of mitochondria and finally causing cell death [17]. It was widely demonstrated that nitrosative stress is associated with OS, inasmuch that some ROS involved in OS act also in the formation and scavenging pathways of nitrogen species. The combination of both OS and nitrosative stress is involved in many different pathologies, including NDDs [18].

**Figure 1:**
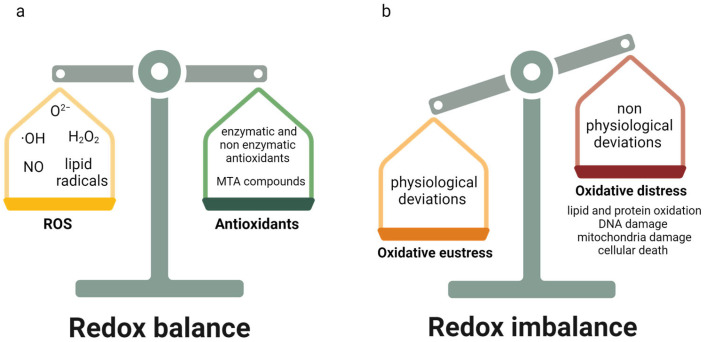
Redox balance and imbalance in organisms. (**a**) The redox balance in living organisms is maintained by the equilibrium between reactive oxygen species (ROS) levels and the action of antioxidants. O^2−^ = superoxide anions, OH = hydroxyl radical, H_2_O_2_ = hydrogen peroxide, NO = nitric oxide, MTA = mitochondria-targeted antioxidant. (**b**) Moreover, both physiological and non-physiological deviations in ROS levels could occur leading to oxidative eustress and oxidative distress, respectively. Oxidative distress causes lipid and protein oxidation, DNA damage, mitochondria damage and finally cellular death (figure created with Biorender.com accessed on 28 January 2024).

There are many different mechanisms, both enzymatic and non-enzymatic, involved in the protection of the organism from the effects of ROS. The principal enzymatic antioxidants include the following: catalase, which is involved in the conversion of H_2_O_2_ to water and oxygen, using manganese or iron as cofactors; superoxide dismutases, which convert reactive O^2−^ to less reactive H_2_O_2_ and oxygen; and glutathione peroxidase, which allows for the reduction of H_2_O_2_ and lipid peroxides. Besides that, non-enzymatic antioxidants include thioredoxin; glutathione; vitamins A, E, and C; flavonoids; proteins; and trace elements [19,20,21] (Figure 1).

Numerous studies pointed out that OS-induced damages are key factors in the aging process and, consequently, in the development of NDDs. Various mitochondrial DNA deletions and a decrease in the number of antioxidants have been found in elderly individuals [22,23,24,25]. For these reasons, it is evident that treatments using antioxidants are fundamental to act against OS in mitochondria. To maintain mitochondrial homeostasis, drugs should accumulate in the mitochondria and interact with their targets. Conventional antioxidants do not accumulate in disease mitochondria, but in recent years, mitochondria-targeted antioxidant (MTA) compounds have been developed [26,27]. These compounds are antioxidant molecules conjugated with a carrier, such as triphenylphosphonium (TPP), which allows for the transport of antioxidants through cellular membranes, thanks to its lipophilicity [28]. They act by interrupting the intramitochondrial cascade caused by OS and lead to apoptosis [27].

Different studies have demonstrated that the treatment using MTA compounds has beneficial effects against mitochondria OS. Among them, mitoquinone, resulted from the conjugation of a triphenylphosphonium carrier and a modified ubiquinone, is the most known, inasmuch as it acts in the scavenging of ROS in the mitochondria and remains active for a long time [29]. MitoVitE, derived from the conjugation of TPP and α-tocopherol, a type of vitamin E, acts by counteracting lipid peroxidation, protecting mitochondria and cells from OS and reducing apoptosis [29,30]. Finally, MitoTEMPO, composed of (2,2,6,6-Tetramethylpiperidin-1-yl)oxyl and TPP, acts by converting mitochondrial superoxide into water [31].

A valide alternative to TPP-targeting is the encapsulation of antioxidants in liposomes. Liposome-encapsulated antioxidants allow for the delivery of antioxidants, such as quercetin, N-acetyl-L-cysteine, and vitamin E, without altering their structures and bioactivity. They enter the cells via micropinocytosis, fusing with the mitochondrial membrane and releasing the antioxidants [26].

## 3. Neuroinflammation

Inflammation is the response of the immune system to adverse factors, including pathogens and toxic molecules. Normally, the inflammatory response acts as a defense mechanism to restore tissue homeostasis. When the tissue is injured, a chemical signaling cascade occurs, activating leukocyte chemotaxis at the site of injury. Leukocytes produce cytokines and induce the inflammatory response, characterized by swelling, heat, redness, pain, and a loss of tissue function [32,33]. It is a complex mechanism with the involvement of different cell types, including macrophages, monocytes, lymphocytes, and mast cells [34]. The inflammation resolution process occurs with a cessation of neutrophils’ action and with a reduction in cytokines’ gradient [32,35]. When immediate inflammatory responses to tissue injury are ineffective, chronic inflammation rises [36].

The term neuroinflammation refers to the inflammatory response in the brain and it is one of the main characteristics of NDDs. It could be defined as the inflammatory response to factors that cause a change in the homeostasis in the central nervous system (CNS) [13]. It was widely demonstrated that the inflammatory response in the brain can cause cell injury and increase the blood–brain barrier permeability, leading to a decrease in its protective role [32]. In brain diseases characterized by inflammation, there is the activation of the brain’s immune cells and microglia, which form the primary innate immunity of brain, with the consequent activation of inflammatory mediators, including cytokines, chemokines, secondary messengers, and ROS [37,38,39]. Moreover, microglia undergo cytoskeletal modifications and changes in receptors’ expression, which allow for its migration toward sites of injury [40]. Curiously, CNS protection and host-organism benefits are the goals of microglial activation and of increased cytokine expression, which are also important for processes such as synaptic pruning and memory consolidation [41,42,43,44,45]. However, persistent, excessive, or amplified microglial activation can result in significant pathogenic alterations [40]. Moreover, it was observed that astrocytes play a critical role in the infiltration of CNS by leukocytes, represented mainly by lymphocytes and mononuclear phagocytes [46].

The inflammatory process is directly linked with OS. ROS can promote the expression of pro-inflammatory genes and, simultaneously, neuroinflammation can stimulate ROS production. In a physiological condition, in which redox balance occurs, the inflammatory response acts as a defense mechanism. On the contrary, under pathological conditions, the redox imbalance causes the activation of inflammatory mechanisms, leading to the secretion of pro-inflammatory molecules and of neoepitopes [46,47]. In parallel, pro-inflammatory cytokines, including interleukines (interleukin 1β and interleukin 6), interferons, and tumor necrosis factor, induce the generation of ROS in non-phagocytic cells, principally by the activation of NADPH oxidase (NOX) [46].

**Figure 2:**
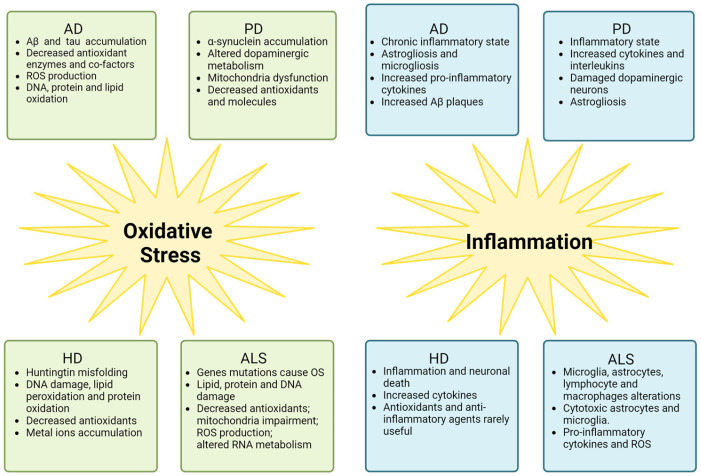
Clinical manifestations of OS and inflammation in four different neurodegenerative diseases (NDDs): Alzheimer’s disease (AD), Parkinson’s disease (PD), Huntington’s disease (HD), and amyotrophic lateral sclerosis (ALS) (figure created with Biorender.com accessed on 28 January 2024).

Both OS and inflammation are involved in NDDs’ pathogenesis, causing common and different manifestations Their involvement is synthesized in Figure 2 and will be discussed in the next paragraphs.

## 4. Oxidative Stress and Inflammation in AD, PD, and HD

AD is the most common type of dementia and it is caused by the loss of cognitive and behavioral capacities, due to the death of neurons of the neocortex, enthorinal cortex, and hippocampus [48]. It is characterized by the accumulation of neurotoxic beta-amyloid (Aβ) oligomer peptides and of tau protein, which causes neuroinflammation, neurotransmitter imbalance, dendritic alterations, and synaptic impairments, all linked to the neurodegeneration [6]. Numerous studies have reported that OS induced by the accumulation of toxic Aβ peptides causes lipids, proteins, and DNA oxidation. Accumulation of Aβ plaques causes different damages, including the disruption of the electron transport chain via cytochrome oxidase inhibition, inevitably leading to OS [48,49,50,51]. Moreover, decreased levels of antioxidant enzymes were found in AD patients [52,53]. An important role of biometals, such as zinc, iron, and copper, was widely demonstrated in AD neurodegeneration, inasmuch as they have been frequently found in Aβ plaques. This causes a deficiency of such metals, important cofactors for antioxidant enzymes in brain cells [54,55,56]. Moreover, Cu^2+^ and Zn^2+^ can bind peptides causing a redox reaction and leading to the production of ROS [57].

Numerous clinical trials have focused on the association between AD and OS, studying the effects of fatty acid supplementation, with some beneficial effects, most of all in cognitive assessment [58,59,60]. For example, it was demonstrated that eicosapentaenoic and docosahexaenoic acids have antioxidant, anti-apoptotic, anti-inflammatory, and neurotrophic properties, enhancing nerve growth factor levels and improving cognitive function [60].

Various studies have demonstrated that AD is characterized by a chronic pro-inflammatory condition in the brain, including both astro- and microgliosis. Aβ-associated depositions cause an increase in pro-inflammatory cytokines’ production by microglia, and, in turn, systemic inflammation enhances β-amyloid generation in the brain [61,62,63]. Moreover, it was recently demonstrated that cytokines produced by microglia, including interleukin 1α, tumor necrosis factor α, and complement component C1q, activate reactive astrocytes involved in neuronal death. Neuroinflammation might prime microglia for such activation [64].

PD is a NDD characterized by the loss of midbrain dopaminergic neurons of the *substantia nigra pars compacta*, causing a reduction of the dopaminergic input to basal ganglia and a hyperactivation of the cholinergic one. The aberrant activation of these pathways contributes to difficulties in memory and learning, and, above all, to the loss of control in motor functions [65].

OS and the overproduction of ROS are important factors involved in degeneration of dopaminergic neurons. The accumulation of ROS was associated with different mechanisms including the metabolism of dopamine itself, mitochondrial dysfunctions in neurons and neuroglia, inflammation, and increased levels of iron and calcium [13,66,67]. It was also observed that neurons of *substantia nigra* accumulate granules of neuromelanin, a pigment which can cause ROS production. Moreover, it was demonstrated that alterations in neuromelanin composition and density can cause α-synuclein aggregation and iron accumulation [68].

Additionally, PD mutations, including the ones on *DJ-1*, *PINK1*, *Parkin*, *SNCA*, and *LRRK2*, have numerous consequences on mitochondrial functions, causing an exacerbation of ROS production [69,70]. With regard to dopamine metabolism, this neurotransmitter is an unstable molecule that is prone to auto-oxidation to form quinones and free radicals. Numerous enzymes are involved in its metabolism and in its degradation by catalyzing its oxidative deamination. However, due to neuronal degeneration, there is an imbalance of these enzymes, which causes the production of ROS [69,70]. Moreover, two enzymes are involved in the defense against ROS, dopamine transporter and vesicular monoamine transporter 2. They both are involved in the uptake of free dopamine from synapses and in its packing into synaptic vesicles to be protected from oxidation. It was demonstrated that dopamine transporter concentration declines with age and that vesicular monoamine transporter 2 is inhibited by α-synuclein, the presynaptic neuronal protein which is found aggregated in PD [68]. Finally, it was proved that in PD patients’ brains, there is a decreased concentration of important antioxidants, such as glutathione and vitamin E, and an alteration in the levels of calcium, iron, and lipids [71,72,73,74,75,76]. As for AD, also for PD, numerous clinical trials have been focused on OS in PD and have studied the deficiency of antioxidants in this pathology and how to ameliorate ROS-caused symptoms. For example, a still ongoing phase II clinical trial is evaluating the effect of the antioxidant N-acetyl cysteine on the dopaminergic function of PD patients [13].

The important role of neuroinflammation in PD was widely demonstrated, mainly due to the activation of microglia and astrocytes. Microglial cells would specifically harm dopaminergic neurons because they are more prevalent in the midbrain than in the other brain areas. It was proved that there was an abundant presence of reactive astrocytes and microglia and an increase in the complement component C1q in the *substantia nigra* of PD-affected subjects, suggesting PD-associated neuroinflammation [77,78,79,80]. Moreover, microglia-activated neuroinflammation mediators, such as cytokines and interleukins, have been detected in the cerebrospinal fluid (CSF) of PD patients [81]. Eventually, the fact that the human leukocyte antigen was found to be a risk factor for PD demonstrated that there is a possibility of a more general pro-inflammatory state in PD, not caused by neuronal loss, although it can worsen the neuroinflammation as well [82].

HD is an inherited NDD that occurs in young individuals. It is a protein-misfolding disease, where the huntingtin protein is mutated and causes an aberration in normal biological functions interacting with other proteins [83]. The mutant protein gains a toxic function leading to OS and inflammation. However, it is still not clear whether OS causes HD or it is a consequence of earlier events and the studies about this aspect are fewer than for other NDDs [84]. Oxidative damage in cells and tissues in HD models and patients has been reported. Lipid peroxidation, protein oxidation, and DNA damage have been linked to this pathology and to huntingtin mutation [85,86,87,88,89,90]. As for the other NDDs, an accumulation of metal ions, especially iron and copper, and a decrease in antioxidant concentrations have been found [91,92,93,94,95]. Moreover, mitochondrial dysfunctions have been detected in HD patients’ brains [96,97]. It was recently demonstrated that the deregulation of HSF1, a transcriptional regulator of the heat shock response, contributes to mitochondrial dysregulation in HD, by impairing the peroxisome proliferator co-activator PGC-1α and its downstream targets such as the mitochondrial transcription factor TFAM and cytochrome c [97]. Neuronal death can activate inflammatory mechanisms, which in turn cause neurodegeneration leading to a vicious cycle [98]. Elevated levels of cytokines have been found in fluids of both animal models and HD patients [99,100,101,102,103]. Unfortunately, until now, anti-inflammatory and antioxidant agents have rarely achieved effectiveness in HD treatment [103].

## 5. Oxidative Stress and Inflammation in ALS

ALS is an NDD which affects the upper and lower motor neurons (MNs) of the cortex, brainstem, and spinal cord, causing the death of patients within three to five years after symptoms’ onset. There are two types of ALS, a sporadic form and a familial one, which can be related to both mutations in specific genes and to epigenetic factors. Despite ALS principal symptoms being related to motor dysfunctions, patients often show signs of behavioral and cognitive impairment [1,104].

There are different factors involved in ALS onset and progression including the presence of OS. In fact, different studies reported OS hallmarks in ALS patients and animal models. Oxidative damage was found in lipids and proteins of post-mortem tissues, as well in plasma, urine, and CSF [105,106,107,108,109]. Moreover, it was demonstrated that nerve terminals are sensitive to ROS and to inflammation, amplifying the decline of neuromuscular junctions [110]. OS was often associated with gene mutations, especially in *SOD1*. *SOD1* catalyzes the conversion of O^2−^ into H_2_O_2_ and molecular oxygen, which are nontoxic for the cells. Specific *SOD1* mutations lead to a higher peroxidase activity and convert H_2_O_2_ to hydroxide, which inactivates the dismutase structure. Furthermore, O^2−^ leads to peroxynitrite production and finally to neuronal death [111]. It is obvious that mutations in this gene can cause serious problems in respiration and the metabolic activities of cells [112]. The gain of function of mutant *SOD1* and aberrant aggregation of this protein were associated with MNs’ death in ALS patients. SOD1 protein aggregation contributes to dysfunction of the ubiquitin/proteasome system and interferes with mitophagy. In addition, mutant *SOD1* leads to impairment in the respiratory chain of mitochondria [113,114,115].

*TARDBP* encodes for the TDP-43 protein, involved in many different processes, including RNA transcription, maturation, transport, and translation. It also participates in intracellular stress management, taking part in the biogenesis and maintenance of stress granules. Mutation in *TARDBP* causes TDP-43 aggregation and cytoplasmic mislocalization, but TDP-43 inclusions were found also in non-mutated patients [111,116]. In turn, aggregated TDP43 causes a mitochondrial imbalance that increases OS [117,118,119]. It was found that OS induces conformation modifications in TDP-43, causing cysteine disulphide cross-linking or promoting lysine acetylation, leading to TDP-43 aggregation and its acquisition of an aberrant function [120,121]. Finally, mutated *TARDBP* decreases antioxidant expression [122,123], influencing the nuclear factor erythroid-2-related factor 2 (Nrf2) antioxidative pathway. TDP43 was also found in stress granules, structures formed in response to stress. In 2011, Dewey and co-authors demonstrated that both wild-type and mutant TDP-43 form stress granules, but mutant TDP-43 sets up larger ones and incorporates them earlier [123,124].

Other common ALS-associated gene mutations include the ones in *FUS* and in *C9ORF72*. *FUS* encodes for an RNA/DNA-binding protein and it is implicated in RNA metabolism and DNA repair. It was demonstrated that *FUS* deficits and mutations fail to repair OS-caused DNA damage due to nick ligation defects, eventually leading to MNs’ death [125]. As for TDP-43, mutations in *FUS* cause mitochondria damage, a decrease in mitochondrial membrane potential and respiration, and a dysregulation in mitochondrial gene transcription [126,127]. In 2020, Tsai and co-authors demonstrated that in *FUS*-mutated cells, FUS protein associates with mitochondria and with mRNAs encoding mitochondrial respiratory chain components. This association causes mitochondrial networks’ disorganization, impairment in aerobic respiration, and an increase in ROS production [126].

Finally, *C9ORF72* is the most commonly mutated gene in ALS patients. In 2016, Onesto and co-authors found that ROS production in *C9ORF72* mutated ALS patients’ cells, causing hyperpolarization of mitochondrial membranes [128]. More recently, Birger and co-authors demonstrated that astrocytes carrying the *C9ORF72* expansion inhibit the production of antioxidant molecules, enhancing OS also in MNs [129]. Additionally, it was found that mutations in this gene increase O^2−^ levels and reduce mitochondrial potential and cell survival [130].

However, signs of OS, including protein and lipid peroxidation, were found in non-mutated ALS cases as well [131,132,133] and were associated with a decrease in antioxidant enzymes’ activity and with a possible pro-oxidative state [134].

As for other NDDs, it is difficult to determine if oxidative damage is a primary cause or a secondary consequence of ALS. Moreover, it is very problematic to evaluate OS markers at an early stage of the disease, ruling out the possibility of evaluating if oxidative damage appears early or late in its course [133]. Nevertheless, animal models have brought some insights into this context. For example, in 2007, Kraft and co-authors obtained a mutant *SOD1* mouse and found an activation of nuclear Nrf2—an antioxidant response element during the disease course. The earliest activation occurred in distal muscles of mice and subsequently caused MN loss [135]. Vande Velde and co-authors arrived at the same conclusions in 2011. They found that mutant *SOD1* causes mitochondria disruption at an early pathogenic stage [136].

The exact oxidative mechanism in ALS is still to be determined, and the involvement of mitochondria in this process is not clear. Moreover, Edaravone, a drug approved in the USA for alleviating ALS symptoms, involved in lipid peroxides and hydroxyl radical elimination, is not particularly effective in disease treatment [137]. In 2019, Walczak and co-authors compared ALS patients and control subjects in terms of mitochondrial function and antioxidant enzymes, and they found a decreased expression in ALS patients’ mitochondria complexes I, II, III, and IV proteins; in mitochondrial membrane potential; and in *SOD1* and catalase, both antioxidant enzymes [138]. In addition, ALS patients carrying mutations in *CHCHD10*, involved in ALS pathology and in mitochondrial cristae morphology maintenance, manifest fibroblasts with mitochondrial damage and mitochondrial network fragmentation [139].

With regard to oxidative DNA damage, 8-hydroxy 2 deoxyguanosine is the most abundant oxidative alteration in DNA, inasmuch as guanine has a low electron reduction potential [137]. Elevated levels of 8-hydroxy 2 deoxyguanosine were found in the motor cortex and spinal cord DNA of ALS patients [140,141]. Furthermore, signs of p53 activation, indicating the apoptosis process, were found in both ALS cellular models and in ALS patients [125,141,142,143]. Altered levels of antioxidant enzymes were found in ALS.

Apurinic/apyrimidinic endonuclease 1 is an enzyme involved in redox regulation and in DNA repair. Its concentration and localization were altered in both ALS patients and animal models [144,145,146]. In addition, alterations in the levels of 8-oxoguanine glycosylase, involved in the removal of oxidized guanine, were found in spinal MNs of both sporadic and mutated ALS cases [144,147].

OS can also bring about abnormalities in RNA metabolism, which in turn can cause OS. It was found that oxidative RNA modifications occur early in the disease progression and precede MN death. Proteins encoded by *TARDBP*, *FUS,* and *SOD1* are involved in miRNA processing and some miRNAs regulate the expression of genes involved in OS [137]. In 2017, Pegoraro and co-authors found an upregulation of both muscle-specific and inflammatory miRNAs in ALS patients compared to control subjects and this upregulation was associated with an earlier age of symptoms onset. Moreover, they found differential miRNA expressions in muscles from males and females, suggesting the influence of sexual hormones [148]. The expression of miR-388-3p, involved in both mitochondrial function and apoptosis, was found increased in *SOD1* mutant mice [149], while the expression of miR-34a, involved in OS regulation, was found decreased in both ALS patients and mouse models [150,151]. MiR-155, involved in inflammatory response and mitochondrial function, was found upregulated in skeletal muscles of ALS patients and in the spinal cord of *SOD1* mice [152,153,154].

Finally, hyperexcitability is a decisive characteristic of ALS, and it was detected before early clinical symptoms, with a strengthening which causes disruption in energy metabolism, mitochondrial disfunctions, and increased OS [155,156,157]. This altered neuronal excitability and the consequent manifestation of OS were associated with defects in ion channels, including sodium, potassium, calcium, and chloride ones, of neuronal and non-neuronal cells [158]. In fact, while most ALS patients do not manifest deleterious mutations in ion channel-associated genes, many studies reported alterations in channels in both mutated and non-mutated ALS subjects and animal models [158,159]. Already in 2006, Kaiser and co-authors demonstrated a reduction in potassium channels in SOD1^G93A^ mice leading inevitably to MN death. More recently, alterations in chloride channels were associated with muscle channelopathies, atrophy, and OS [160,161] and the decreased expression of calcium channels in the spinal MNs of SOD1^G93A^ mice was correlated with excess mitochondrial calcium and the production of ROS [162,163].

Noteworthy, Riluzole acts by blocking voltage-gated channels, especially sodium ones, allowing for the inhibition of glutamate release in presynaptic terminals and interfering with the excitatory transmission caused by this amino acid [164].

With regard to the inflammatory response, alterations in the immune system can cause an increase in neuroinflammation in ALS patients which has been associated with neuronal loss in both animal models and in humans [134]. Alterations were observed in all the cell types involved in inflammation, including microglia, astrocytes, lymphocytes, and macrophages. Activated microglia were found in both ALS patients and animal models, and they were correlated with MN deficits [165,166,167]. The loss of function of *C9ORF72* causes alterations in microglia, macrophages, and neuroinflammation [168]. In addition, different studies have demonstrated a defective lysosomal system with the accumulation of innate immune cells in *C9ORF72* mutated mice [169,170,171].

Many studies have demonstrated that microglia activation and their switch from a protective phenotype to a deleterious one can be mediated by the receptor P2X7, the inhibition of which may provide positive outcomes in ALS patients [172,173,174,175]. Furthermore, injured MNs induce microglia to acquire a cytotoxic phenotype with the consequent release of ROS and pro-inflammatory cytokines [176,177,178,179]. Among them, interleukin 6 was correlated with disease progression in ALS [180]. A more recent study tested a cohort of 53 ALS patients through positron emission tomography. Authors used [11C]-PBR28, a radiotracer that binds to a protein typically expressed in activated microglia, and found that glial activation is increased in the pathological brain region and is correlated with clinical measures [181].

Even mutated astrocytes are toxic to normal MNs, causing their death [182,183,184]. In 2017, Qian and co-authors demonstrated that both non-MNs and MNs degenerate after ALS astrocyte transplantation, suggesting that neural degeneration is not specific to MNs and that the astrocyte-mediated neuronal death occurs through a non-cell autonomous toxicity [185]. Moreover, ALS causes the loss of glutamate transporter on astrocytes, responsible for the uptake of excess glutamate from synaptic clefts. It was demonstrated that the inefficient glutamate uptake exacerbates MN degeneration [186,187]. As well as microglia, astrocytes exert toxic effects on MNs by secreting pro-inflammatory molecules, including NO, NOX2, prostaglandin E2, and leukotriene B4, or inducing necroptosis [188,189,190,191].

In addition to astrocytes and microglia, dysregulation in T lymphocytes and macrophages was observed in ALS patients and ALS animal models [192,193,194,195,196,197]. In 2020, Chiot and co-authors demonstrated that the replacement of macrophages in *SOD1* mice by more neurotrophic macrophages led to a decrease in macrophage and microglia activation. Moreover, they found that when the replacement occurs in pre-symptomatic stages, it causes a delay in disease onset, whereas when it occurs at the disease onset, it is able to increase mice survival [198].

## 6. ALS Therapeutic Approaches Related to OS and Inflammation

Several trials to find therapies for ALS have been conducted or are still on course, and some of them are related to **OS**. In this regard, one of two drugs approved in the USA for the treatment of ALS, Edaravone, acts as a **scavenger of ROS**, thus preventing OS propagation [199,200]. In a recent study, Ohta and co-authors demonstrated that in the CSF and plasma of ALS patients, there is a reduction in antioxidant capacities, measured using the OXY-adsorbent test, which is reversed by Edaravone treatment [201]. The other approved drug, Riluzole, blocks glutamatergic neurotransmission and inhibits glutamate release. Different studies have proved that it can also attenuate **OS** injuries in in vitro and in vivo ALS models [202,203]. However, the exact mechanism of action of Riluzole is still unknown.

Studies have been performed also on molecules which upregulate genes containing the antioxidant response element. Among them, sulforaphane activates the Nrf2/**antioxidant response** element pathway but did not show effects in ALS treatment [204,205]. A limitation in the use of sulforaphane could be that the combination of sulforaphane and antioxidants reduces the protective effects of sulforaphane itself, specifically in the induction of autophagy [206]. Moreover, further studies have pointed out possible side effects of sulforaphane, which can induce a lowering of the seizure threshold in mice [207] and can influence thyroid activity [208]. More recently, CuATSM, a positron emission tomography-imaging agent which is able to deliver copper to cells with altered mitochondria, has been proved to extend the survival and to delay ALS onset in SODG93A mice, acting on **OS response** [209,210]. However, it is not tolerated at a high dose in mice, causing toxicity signs including motor aberrations, weight loss, and low activity [190].

Studies have also focused on mitochondrial-targeting drugs, such as mitoquinone and Szeto-Schiller peptides, which act by **decreasing oxidative damage** and maintaining normal mitochondrial function [111,211]. Additionally, the p62-mediated mitophagy inducer was seen as a promoter of the quality control of mitochondria and an inducer of autophagy in damaged organelles, without evident adverse effects, acting as a potential ALS therapeutic molecule [212,213].

Other promising approaches to **reduce OS** are the use of phytochemicals, such as quercetin, which decreases ROS in *SOD1* mutated cells [214] but could, however, have possible nephrotoxic effects and interact with other drugs [215], and the use of cannabidiol or the target of cannabinoid receptors [216,217,218]. Finally, many studies have suggested the possibility to use **modifiers of OS-related molecules**, such as NOX inhibitors. NOX activity is unregulated in ALS patients and animal models, causing inflammation and glial activation. It was observed that the use of NOX inhibitors, such as perphenazine, thioridazine, and apocynin, reduces O^2−^ levels, increases the numbers of MNs, and extends the lifespan, but it could have also sedative effects [219,220].

Despite different approaches, finding a definitive drug is difficult, because the direct pathogenic mechanism of ALS is not clear. One promising option would be the design of **antioxidant therapies** also **associated with anti-inflammatory therapeutics** [110]. Multiple **anti-inflammatory compounds** have been tested and have been shown to be effective for ALS treatment, especially in animal models. Minocycline was tested on ALS animal models, demonstrating a high efficacy in reducing MN loss, extending mice survival, and suppressing microglia activation [221,222]. However, a phase III trial on 412 ALS patients revealed harmful effects, including gastrointestinal, respiratory, and neurological ones, without significant results on disease progression [223]. On the contrary, a recently concluded phase II trial on NP001, a regulator of macrophage activation, revealed the good tolerability of this compound, leading to the slowing of ALSFRS-R and vital capacity scores in a subgroup of treated patients [224,225]. The only side effect reported was higher infusion-related sensations of burning [225]. Better results were obtained with the use of masitinib, a tyrosine-kinase inhibitor, that is able to decrease aberrant glial cells, microgliosis, and MN degeneration in mice [226]. The clinical trial on ALS patients demonstrated a slowing in functional decline in patients and a prolonged survival by over two years, with the most common side effects including maculopapular rash and peripheral edema [227,228].

In the field of drug repurposing, Fingolimod, a modulator of sphingosine-1-phosphate receptor approved for the treatment of relapsing remitting multiple sclerosis, was tested on *SOD1* mutated mice and was observed to act on **inflammation**, having beneficial effects modulating microglia and innate immunity and reducing the levels of inducible NO synthase [229]. In a recent phase II trial, Fingolimod resulted as well tolerated by ALS patients and showed the possibility to reduce circulating lymphocytes, with no serious adverse events [230]. Moreover, it was demonstrated that Fingolimod acts also on **OS**, reducing the levels OS markers and increasing antioxidants, especially SOD [231,232]. A recent study by Yevgi and Demir tested the action of this drug on multiple sclerosis patients and demonstrated a reduction in the total OS after three months of treatment [233].

Other studies focused on the use of ***immune modulatory drugs***. In a pilot trial of 2019, authors treated ALS patients with RNS60, an immune-modulatory agent, demonstrating its safety and tolerability, with common adverse effects including falls, headaches, nasopharyngitis, and contusions and no serious adverse effects [234]. More recently, a trial targeted T cells with a low dose of interleukine 2, demonstrating a high tolerability and an immunologically efficacy in ALS patients, with an increase in Treg levels. No serious adverse events were reported and the non-serious adverse effects were transient [235]. Moreover, numerous studies have focused on cell-based treatments, especially for the use of mesenchymal, embryonic, and neural progenitor cells [236,237].

Finally, it was demonstrated that the regular use of anti-hypertensive drugs could have a protective role against ALS incidence. Hypertension is one of the most common comorbidities in ALS and it was related to progression, incidence, and survival, mainly for the involvement of angiotensin 2 in ROS production. Angiotensin 2 is a potent stimulator of NAD(P)H oxidase, one of the major sources of ROS. ROS generated by NAD(P)H oxidase can induce the production of other ROS and could lead to inflammation [238,239,240,241]. In 2020, Pfeiffer and co-authors demonstrated that numerous hypertension drugs, including beta blockers, angiotensin-converting enzyme inhibitors, calcium channel blockers, diuretics, and angiotensin 2 receptor blockers, were correlated with a **lower risk of ALS** [242]. Moreover, it was demonstrated that the angiotensin system can be involved in different NDDs and that its blocking can be a new method for **neuroprotection** [243,244,245].

These studies demonstrated that many drugs tested are effective in animal models but not in clinical practice. This gap can be explained by the complexity of the ALS disease mechanism and by the fact that preclinical models could not completely recapitulate the disease processes which occur in humans [246.

The still ongoing clinical trials, testing the efficacy of the abovementioned drugs, are reported in Table 1.

## 7. Conclusive Remarks

OS and inflammation are important mechanisms involved in NDDs and, among them, in ALS pathology. They can be caused by external or internal triggers, such as chemicals and ROS-developing enzymes, respectively, but they always lead to pathological aberrations, including DNA and RNA damage, mitochondrial dysfunction, and cell death. Despite many studies focused on their role in neurological diseases, uncertainty still exists over whether they contribute to the development of NDDs or are solely a consequence of neuronal degeneration. This review summarized the recent findings on the involvement of these two pathological pathways in NDDs, with a specific focus on ALS. We highlighted that all the discussed NDDs, i.e., AD, PD, HD, and ALS, are characterized by an oxidative and inflammatory state which leads to similar pathological mechanisms, including cell damage, lipid and protein oxidation, DNA aberrations, and finally neuronal death. Moreover, we underlined how these two mechanisms are intrinsically correlated in a vicious cycle. The generation of ROS causes neuronal damage and the release of molecules that activate microglia and astrocytes. In turn, these cells release pro-inflammatory cytokines which cause inflammation and exacerbate neuronal injury.

Finally, we discussed some evidence of possible therapeutic approaches targeting OS and the inflammation pathway for the treatment of ALS. As for the other NDDs, many studies focused on the use of antioxidants and anti-inflammatory compounds for the treatment of this still incurable pathology, but few drugs, including NP001 and Fingolimod, have shown efficacy in humans especially for the multifactorial characteristic of these diseases. Although the finding of a definitive drug is difficult and there is a need for future studies, there are numerous clinical trials which will deepen the knowledge about these diseases and will elucidate the precise mechanisms underlining OS and the inflammatory response.

## Figures and Tables

**Table 1:** Ongoing clinical trials. List of the still ongoing clinical trials of the mentioned drugs (https://clinicaltrials.gov accessed on 29 January 2024).

Drug	Action Mechanism of the Drug	Name of the Trial	Clinical Trial ID	Clinical Trial Phase
Edaravone	Scavenger of ROS	Study to investigate the efficacy and safety of FAB122 (daily oral Edaravone) in patients with amyotrophic lateral sclerosis	NCT05178810	Phase III
		Radicava^®^ (Edaravone) Findings in Biomarkers from ALS (REFINE-ALS)	NCT04259255	Phase IV
Riluzole	Glutamatergic neurotransmission blocking	Treatment combining riluzole and IFB-088 in bulbar amyotrophic lateral sclerosis (TRIALS protocol)	NCT05508074	Phase II
Cannabidiol and cannabinoids	OS reducing	Outcomes Mandate National Integration with Cannabis as Medicine (OMNI-Can)	NCT03944447	Phase II
		Safety and efficacy on spasticity symptoms of a cannabis sativa extract in motor neuron disease	NCT01776970	Phase II and phase III
		Efficacy of Cannabinoids in Amyotrophic Lateral Sclerosis or Motor Neurone Disease	NCT03690791	Phase III
		EMERALD TRIAL Open Label Extension Study (EMERALD-OLE)	NCT04997954	Phase IV
Masitinib	Anti-inflammatory compounds	Efficacy and Safety of Masitinib Versus Placebo in the Treatment of ALS Patients	NCT03127267	Phase III
RNS60	Immune modulatory drug	Nebulized RNS60 for the Treatment of Amyotrophic Lateral Sclerosis	NCT02988297	Phase II not yet recruiting
Stem cells	Immune system modulator	The Evaluation of the Effect of Mesenchymal Stem Cells on the Immune System of Patients with ALS (ALSTEM)	NCT04651855	Phase I and II
		Derivation of Induced Pluripotent Stem Cells from an Existing Collection of Human Somatic Cells	NCT00801333	Observational
		CNS10-NPC-GDNF Delivered to the Motor Cortex for ALS	NCT05306457	Phase I
		Neurologic Stem Cell Treatment Study (NEST)	NCT02795052	Interventional
		Development of iPS From Donated Somatic Cells of Patients with Neurological Diseases	NCT00874783	Interventional
Calcium channel blockers	ROS reducer	Rho Kinase Inhibitor in Amyotrophic Lateral Sclerosis (REAL)	NCT05218668	Phase II
